# Variability in aminoglycoside dosing in intensive care units across London - should our methods be standardised?

**DOI:** 10.1186/2197-425X-3-S1-A400

**Published:** 2015-10-01

**Authors:** JM Singleton, KE Grailey, JB Simon, JLC Wong

**Affiliations:** Imperial College London, London, United Kingdom

## Introduction

Aminoglycoside doses are optimised to ensure adequate peak levels are attained for the desired bactericidal effect. Dose adjustments are increasingly realised to be important in the critical care setting where pharmacokinetics of agents may vary [[1]]. Variables affecting dose given include patient weight, renal function, dose per unit weight and subsequent re-dosing schedule. It is currently unclear if any dosing regime is superior.

## Objectives

We assessed the availability of different aminoglycosides in Intensive Care Units (ICUs) across London. We compared the dosing and subsequent re-dosing regimes for gentamicin, and surveyed the protocols for aminoglycoside use in renal replacement therapy (RRT).

## Methods

A 7-part questionnaire was developed, and data collection was achieved by telephone interviews with Critical Care Pharmacists at 27 London Hospitals between February and April 2015.

## Results

All 27 units had access to gentamicin with 23 (85%) of the units using it as their first line aminoglycoside. 26 of the 27 units also had access to amikacin but only 11 hospitals reported its use as regular.

The most common initial dosing regimen for gentamicin was 5mg/kg. Five ICUs (19%) used a higher initial dosing regime of 7mg/kg.

Corrected body weight (CBW) calculations were employed by 26 of the 27 ICUs to dose gentamicin for obese patients. 19 ICUs defined obesity as an actual body weight (ABW) of greater than 20% of patients' IBW whilst 4 ICUs used a cut off of 15%. 3 ICUs defined obesity using the body mass index (BMI) scale.

Maximum initial gentamicin doses ranged from 450mg to 600mg and 5 ICUs had no maximum defined dose. 20 of the ICUs (75%) used trough levels to guide gentamicin re-dosing and the remaining 7 (25%) used a nomogram.

14 ICUs (52%) had no specific protocols to guide dosing for patients undergoing RRT. 10 ICUs (37%) dosed these patients with a defined dose per kilogram (ranging from 3 to 7mg/kg) and then used trough levels to guide re-dosing. 2 units (7%) used three times a day dosing and one unit reported they avoided gentamicin in RRT.

## Conclusions

There is a large variation in the dose, maximum dose and re-dosing of gentamicin across ICUs. Those patients undergoing RRT have a wide variability in the total dose that could potentially be administered. Further work must be done to determine whether there are correlations between the different dosing regimes and outcomes. This preliminary work has highlighted the current aminoglycoside dose variability and we intend to further this work by extending it across the United Kingdom.
